# Effects of UV-B Radiation Exposure on Transgenerational Plasticity in Grain Morphology and Proanthocyanidin Content in Yuanyang Red Rice

**DOI:** 10.3390/ijms25094766

**Published:** 2024-04-27

**Authors:** Lin Zhang, Xiupin Wang, Yanqun Zu, Yongmei He, Zuran Li, Yuan Li

**Affiliations:** 1 College of Resources and Environment, Yunnan Agricultural University, Kunming 650201, China; 2 College of Faculty of Animal Science and Technology, Yunnan Agricultural University, Kunming 650201, China; 3 College of Horticulture and Landscape, Yunnan Agricultural University, Kunming 650201, China

**Keywords:** UV-B, transgenerational plasticity, proanthocyanidins, rice, grain morphology

## Abstract

The effect of UV-B radiation exposure on transgenerational plasticity, the phenomenon whereby the parental environment influences both the parent’s and the offspring’s phenotype, is poorly understood. To investigate the impact of exposing successive generations of rice plants to UV-B radiation on seed morphology and proanthocyanidin content, the local traditional rice variety ‘Baijiaolaojing’ was planted on terraces in Yuanyang county and subjected to enhanced UV-B radiation treatments. The radiation intensity that caused the maximum phenotypic plasticity (7.5 kJ·m^−2^) was selected for further study, and the rice crops were cultivated for four successive generations. The results show that in the same generation, enhanced UV-B radiation resulted in significant decreases in grain length, grain width, spike weight, and thousand-grain weight, as well as significant increases in empty grain percentage and proanthocyanidin content, compared with crops grown under natural light conditions. Proanthocyanidin content increased as the number of generations of rice exposed to radiation increased, but in generation G3, it decreased, along with the empty grain ratio. At the same time, biomass, tiller number, and thousand-grain weight increased, and rice growth returned to control levels. When the offspring’s radiation memory and growth environment did not match, rice growth was negatively affected, and seed proanthocyanidin content was increased to maintain seed activity. The correlation analysis results show that phenylalanine ammonialyase (PAL), cinnamate-4-hydroxylase (C4H), dihydroflavonol 4-reductase (DFR), and 4-coumarate:CoA ligase (4CL) enzyme activity positively influenced proanthocyanidin content. Overall, UV-B radiation affected transgenerational plasticity in seed morphology and proanthocyanidin content, showing that rice was able to adapt to this stressor if previous generations had been continuously exposed to treatment.

## 1. Introduction

Ultraviolet-B (UV-B) radiation (280–315 nm) is a biologically potent form of radiation whose increase due to the depletion of the atmospheric ozone layer can affect plants. Although the Montreal Protocol and its amendments set out measures aimed at protecting the Earth’s surface layer from UV radiation, it is uncertain how surface UV radiation may change in the future [1]. The triple interaction of stratospheric ozone, UV-B radiation, and climate change is projected to increase the UV index in the tropics and at mid-latitudes by 3–8% by 2100 [2]. Radiation in the environment exerts a dual effect on plants, acting as both a special regulator and an environmental stress factor. Regarding the former, studies have shown that moderate UV-B radiation exposure promotes plant growth and regulates metabolism and that low-dose gamma radiation pretreatment enhances plant stress tolerance [3]. On the other hand, excessive UV-B radiation, as a stress factor, can hinder plant growth [4]. The accumulation of ‘UV-B sunscreen’ flavonoid substances, plant dwarfing, greater branching leaf reduction, and cell wall thickening are specific response mechanisms employed by plants to adapt to UV-B radiation. Proanthocyanidins are UV-absorbing substances that shield plant tissues from penetrating radiation and have very strong antioxidant ability; therefore, they are among the central factors of a plant’s adaptive response to radiation at this wavelength [5].

Plant phenotypes are not only genetically determined but also influenced by environmental factors. Phenotypic plasticity, i.e., the change in the phenotype of a genotype in response to different environments, is considered a mechanism by which plants can adapt to environmental changes [6]. Transgenerational plasticity, the phenomenon whereby the phenotype of the plant offspring is influenced by the parental environment [7], is achieved through various mechanisms, including epigenetic regulation, such as DNA methylation and histone modifications, and the activity of hormones, metabolites, or defense proteins [8]. Mother plants store environmental cues in seeds in these ways, influencing their phenotypes. Currently, evidence for six different transgenerational patterns, including rebound, attenuation, persistence, accumulation, delay, and reversal, has been found [9]. In plants, environmental stress induces transgenerational plasticity, and relevant factors include temperature [10], drought [11], heavy metals [12], and radiation [13]. Similarly, salinity [14] and mineral deficiencies [15] can also affect plant growth, and attention should be paid to transgenerational plant plasticity induced by these changes. It has been reported that greater quantities of defense substances were allocated to younger isolates of *Glechoma longituba* L. mother plants after exposure to high-frequency UV-B radiation [16]. Long-term exposure of *Vicia faba* L. to short solar ultraviolet (UV) light exerted transgenerational effects on the morphology and flavonoids of the progeny, as observed when the latter were subjected to blue light and UV-B [17]. Further, UV-B-induced physiological, epigenetic, and, specifically, DNA methylation changes have been reported to affect gene expression in progeny. These studies validate that transgenerational plasticity helps plants cope with exposure to a UV-B stress environment in successive generations.

Transgenerational plasticity is based on stress memory, which helps plants adapt to environmental stress when the parental environment is highly predictive of the offspring’s environment [18]. For example, in potato, water stress memory enhances drought tolerance and antioxidant activity and increases yield in some varieties under non-limiting water conditions [19]. The memory effect depends on the genotype of the plant, as well as on whether the scale of observation is physiological, biochemical, metabolic, or molecular. Therefore, studies of phenotypic plasticity must consider these organizational levels [20]. Offspring adaptation can also be negatively affected by parental stress memory. For instance, elevated-CO_2_ environments induce transgenerational effects on stomatal density and yield reduction in rice [21], and *Gossypium hirsutum* L. drought memory causes progeny seed germination to slow down. Therefore, in the absence of stress signals, transcriptional memory relative to a specific stressor can only be maintained for a short period of time, after which it disappears [22], prioritizing the recording of responses to other stressors in order to achieve higher environmental adaptability.

Proanthocyanidins serve as an important UV-B radiation shielding material, as when present in increased levels, these compounds can filter ultraviolet-B radiation from sunlight while acting as physiological antioxidants capable of scavenging free radicals and slowing cellular damage caused by radiation at this wavelength [23]. UV-B radiation affects metabolism and gene expression, regulating flavonoid metabolism and promoting proanthocyanidin synthesis [24]. For example, in a previous research study, UV stress effectively increased proanthocyanidin content and total phenols, and correspondingly, OH· and ABTS scavenging capacity was improved [25]. In another study, the authors found a correlation between the accumulation of flavonols and hydroxycinnamic acid in the berries and fruits of *Vitis vinifera* L. plants, respectively, that had been treated with UV-B irradiation for the previous two growing seasons [26]. However, it is unclear whether transgenerational plasticity in proanthocyanidin content is induced when plants are exposed to UV-B radiation.

Rice is one of the three major cereals, representing 46% of the world’s total cereal supply. Proanthocyanidins are color-presenting substances in red rice kernels that reduce UV-B radiation damage. Rice seed morphology also affects rice thousand-grain weight and yield [27]. The Yuanyang terraces in Yunnan Province, China, form a high-altitude, sustainable rice field agro-ecosystem. ‘Baijiaolaojing’ is a local, traditionally planted red rice variety that meets the requirements for transgenerational genetic research, as its high degree of environmental adaptation compensates for the lack of hybrid rice varieties that differentiate immediately after planting. Therefore, in this study, we chose Baijianglaojing as the test material and exposed the crops to enhanced UV-B radiation in situ to study the changes in red rice kernels and transgenerational plasticity in proanthocyanidin content in four generations of red rice under UV-B stress. The aim was to be able to predict the effects of UV-B radiation exposure on rice growth and consequently formulate appropriate interventions to mitigate them.

## 2. Results

### 2.1. Effects of UV-B Radiation Exposure on Rice Seeds and Proanthocyanidin Content

UV-B radiation exposure at different intensities affected rice spike, grain morphology, and proanthocyanidin content in rice generation G1 (Table 1). As the radiation intensity increased, the spike weight, grain length, grain width, and thousand-grain weight of rice decreased to different degrees. Spike weight decreased by 15.9% under 2.5 kJ·m^−2^ radiation compared with natural light (0 kJ·m^−2^). Under 7.5 kJ·m^−2^ radiation, grain length, grain width, and thousand-grain weight significantly decreased to the lowest level by 14.8%, 8.0%, and 14.2%, respectively, compared with plants under natural light. However, UV-B radiation at different intensities did not significantly affect spike length in this crop. The higher the intensity of UV-B radiation was, the more significant the effect on the empty grain ratio was, where the highest rate was 17.13% at 7.5 kJ·m^−2^, an increase of 104.4% compared with natural light conditions. Proanthocyanidin content in seeds increased with the increase in the intensity of UV-B radiation, reaching a maximum under 5 kJ·m^−2^ but decreasing under 7.5 kJ·m^−2^. In generation G1, the plasticity response index improved with the radiation intensity. *PPI*_G1_ (i.e., generation G1’s phenotypic plasticity index; detailed calculations can be found in Section 4.4) reached its maximum value at 7.5 kJ·m^−2^ when plasticity was high.

### 2.2. Effect of UV-B Radiation on Transgenerational Plasticity in Rice

#### 2.2.1. Transgenerational Plasticity and Rice Growth

**Figure 1:**
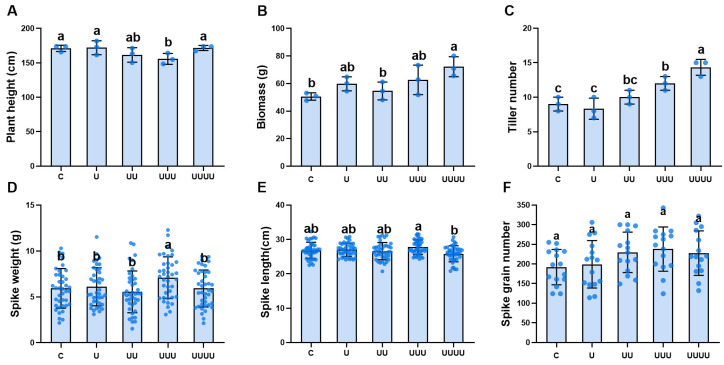
The effects of UV-B radiation exposure on growth in different generations of rice. For the four generations, (**A**–**F**) indicate plant height, biomass, tiller number, spike length, spike weight, and number of grains per spike, respectively. Generations U, UU, UUU, and UUUU represent rice plants that were continuously exposed to UV-B radiation for 1, 2, 3, and 4 years, respectively. C represents the control, i.e., plants that did not experience UV-B radiation treatment but were kept under natural light. Different lowercase letters indicate significant differences among treatments (according to one-way ANOVA with the Duncan test; *p* < 0.05). The data are means ± SEs (plant height, biomass, and tiller number: n = 3; spike length and spike weight: n = 40).

Based on the plasticity response index of rice in generation G1 (*PPI*_G1_) under different UV-B radiation intensities, the radiation treatment with the largest value, i.e., the 7.5 kJ·m^−2^ treatment, was selected for further experiments, and the transgenerational impacts of UV-B radiation exposure on rice offspring (G1, G2, G3, and G4) were examined. Under the selected treatment, rice plant height showed a tendency to decrease and then increase across generations exposed to radiation compared with natural light. This parameter decreased to its lowest value, 155.8 cm, in G3; then, in G4, it increased significantly to 171.5 cm, an increase of 10.0%, thus returning to the control level (Figure 1A). In G4, biomass and tiller number reached their maximum values. The most significant change was observed in the latter, which increased significantly by 59.2% compared with the control (Figure 1B,C). Spike length decreased with the increase in generations of rice exposed to UV-B radiation, but it was significantly higher in G3 than in G2 (Figure 1D). Spike weight increased significantly only in G3 (Figure 1E). The exposure to UV-B radiation of successive generations increased the spike grain number to some extent, but there were no statistical differences (Figure 1F).

#### 2.2.2. Transgenerational Plasticity in Rice Grain Morphology

**Figure 2:**
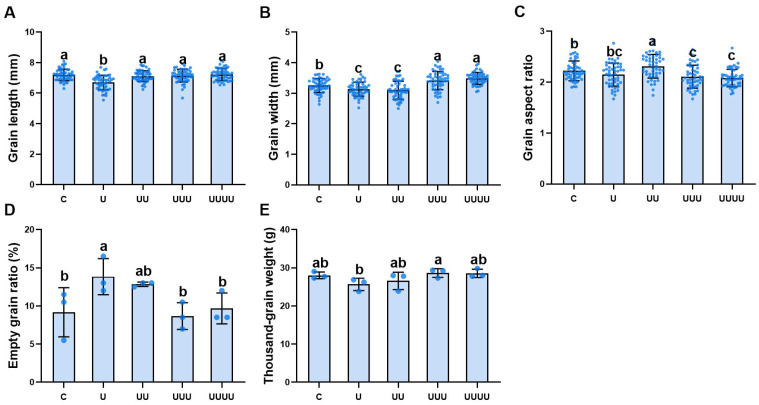
The effects of UV-B radiation exposure on grain morphology in different generations of rice. For the four generations, (**A**–**E**) indicate grain length, grain width, grain aspect ratio, empty grain ratio, and thousand-grain weight, respectively. Generations U, UU, UUU, and UUUU represent rice plants that were continuously exposed to UV-B radiation for 1, 2, 3, and 4 years, respectively. C represents the control, i.e., plants that did not experience UV-B radiation treatment but were kept under natural light. Different lowercase letters indicate significant differences among treatments (according to one-way ANOVA with the Duncan test; *p* < 0.05). The data are means ± SEs (grain length, grain width, grain aspect ratio: n = 50; empty grain ratio, thousand-grain weight: n = 3).

Under treatment at a radiation intensity of 7.5 kJ∙m^−2^, grain length, grain width, and thousand-grain weight all decreased and then increased in radiation-exposed generations compared with the control and decreased significantly compared with G1. Grain length and thousand-grain weight returned to the control levels in G2, while grain width increased significantly from G3 onwards (Figure 2A,B,E). The changes in grain length and width resulted in a significant increase in grain aspect ratio in G3 (Figure 2C). The empty grain ratio first increased and then decreased over the generations, with a changing trend opposite to that of thousand-grain weight; specifically, it was at its maximum in G1, exhibiting a significant increase of 50.9% compared with the control, and it decreased significantly in G3 (Figure 2D).

#### 2.2.3. Transgenerational Plasticity in MDA/Proanthocyanidin Contents

**Figure 3:**
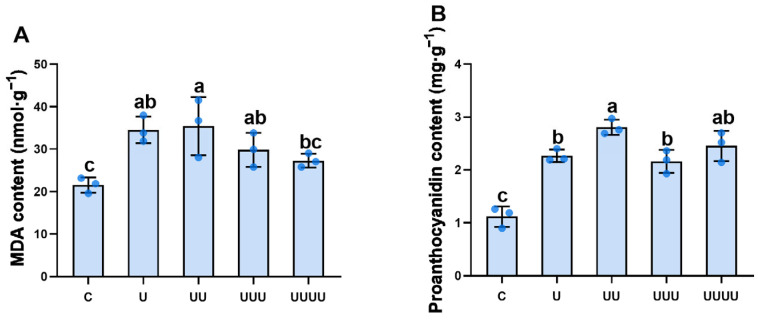
The effects of UV-B radiation exposure on MDA and proanthocyanidin contents in different generations of rice. For the four generations, (**A**,**B**) indicate MDA and proanthocyanidin contents. Generations U, UU, UUU, and UUUU represent rice plants that were continuously exposed to UV-B radiation for 1, 2, 3, and 4 years, respectively. C represents the control, i.e., plants that did not experience UV-B radiation treatment but were kept under natural light. Different lowercase letters indicate significant differences among treatments (according to one-way ANOVA with the Duncan test; *p* < 0.05). The data are means ± SEs (MDA and proanthocyanidin contents: n = 3).

Under treatment at the radiation intensity of 7.5 kJ∙m^−2^, rice kernel malondialdehyde (MDA) content initially significantly increased in the radiation-exposed generations, reaching its maximum in G2, but declined after G3 and returned to the control level (Figure 3A). Rice proanthocyanidin content increased and then decreased over the generations, with the highest content being observed in G2, representing a significant increase of 150.9% compared with the control. This parameter then decreased significantly in G3 and G4 but remained higher than in the control (Figure 3B).

#### 2.2.4. Transgenerational Plasticity in Proanthocyanidin Synthase

The exposure of successive generations of rice to UV-B radiation significantly affected key proanthocyanidin synthesis enzymes. Compared with the control, phenylalanine ammonialyase (PAL), cinnamate-4-hydroxylase (C4H), dihydroflavonol 4-reductase (DFR), and 4-coumarate:CoA ligase (4CL) enzyme activity were significantly increased in G1 and reached maximum values in G2. Anthocyanidin reductase (ANR) activity also increased but not significantly. In the subsequent generation (G3), PAL and C4H enzyme activity were significantly decreased. In contrast, ANR and 4CL activity declined significantly only in G4, returning to control levels. DFR activity, although it declined significantly in G3, increased in G4. In particular, UV-B radiation inhibited chalcone isomerase (CHI) activity, and the exposure of successive generations to UV-B radiation also caused a gradual decrease in CHI activity, reaching a minimum in G4.

**Figure 4:**
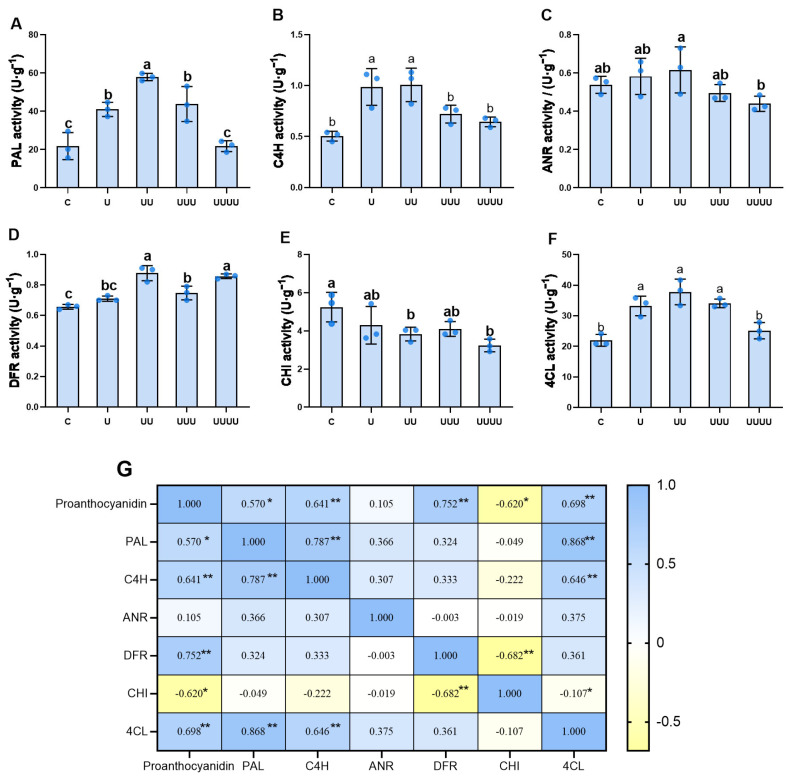
The results of the correlation analysis between proanthocyanidin content and enzyme activity. (**A**–**F**) represent PAL, C4H, ANR, DFR, CHI, and 4CL activity, respectively, in four generations of rice. U, UU, UUU, and UUUU represent generations G1, G2, G3, and G4, respectively. C represents the control, i.e., plants that did not undergo treatment with UV-B radiation but were kept under natural light. Different lowercase letters indicate significant differences between treatments (according to one-way ANOVA with the Duncan test; *p* < 0.05). The data are means ± SEs (n = 3). (**G**) represents the correlation between proanthocyanidin content and enzyme activity (according to Pearson’s correlation analysis); * indicates a significant correlation (*p* < 0.05) and ** indicates an extremely significant correlation (*p* < 0.01).

The correspondence analysis showed that PAL, C4H, DFR, and 4CL activity were significantly positively correlated with proanthocyanidin content, while CHI activity was significantly negatively correlated with it (Figure 4G).

### 2.3. Effects of UV-B Stress Memory on Rice Offspring

#### 2.3.1. Memory Effect on Offspring Growth

When the parent develops stress memory following exposure to enhanced UV-B radiation, if the offspring also receive the same treatment, then the transgenerational memory matches the environment; if, on the other hand, the offspring receive natural light treatment, then there is a memory–environment mismatch.

**Figure 5:**
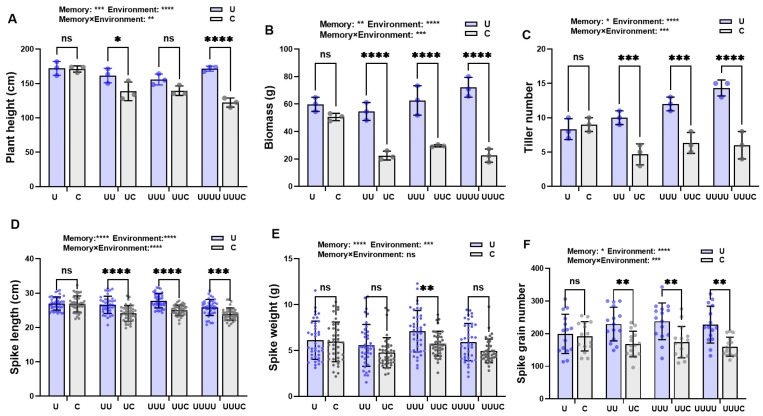
The effects of transgenerational plasticity on growth in different generations of rice. (**A**–**F**) represent plant height, biomass, tiller number, spike length, spike weight, and spike grain number under the two treatments in different generations Purple bars represent that the current radiation environment is UV-B and gray bars represent that the current radiation environment is natural light. The horizontal coordinates indicate the different generations (G1, G2, G3, and G4). C and U correspond to generation G1 (where C: natural light treatment, i.e., control; U: one-year UV-B radiation treatment), UC and UU correspond to G2 (where UC: one-year UV-B radiation treatment followed by one-year natural light treatment; UU: two-year continuous UV-B radiation treatment), UUC and UUU correspond to G3 (where UUC: two-year UV-B radiation treatment followed by one-year natural light treatment; UUU: three-year continuous UV-B radiation treatment), and UUUC and UUUU correspond to G4 (where UUUC: three-year UV-B radiation treatment followed by one-year natural light treatment; UUUU: four-year continuous UV-B radiation treatment). * indicates a significant effect (*p* < 0.05), ** indicates a highly significant effect (*p* < 0.01), *** indicates highly significant difference (*p*<0.001), **** indicates very highly significant difference (*p*<0.0001), and ns indicates no significant effects (according to two-way ANOVA).

Within the same generation of rice plants possessing radiation memory, plant height, biomass, tiller number, spike length, and number of grains in a spike were significantly decreased under natural light exposure (radiation memory–environment mismatch) compared with UV-B radiation exposure (Figure 5A–D,F). However, spike weight was significantly decreased only in G3, and no significant differences were observed in any of the other generations (Figure 5E). Plant height, tiller number, and biomass also decreased over the generations under memory–environment mismatch, implying that the older the generation in which the stress memory originated, the more obvious the growth disruption. In contrast, when memory and environment matched, growth gradually returned to control levels; most notably, biomass and tiller number increased across the generations (Figure 5B,C).

Based on radiation memory and environmental radiation intensity, two-way ANOVA was performed to investigate growth parameters in the four generations of rice. Radiation memory and environment had significant effects on plant height, biomass, tiller number, spike length, spike weight, and spike grain number but not on spike weight. Memory–environment interactions affected rice growth, but no significant effect on grain weight was recorded.

#### 2.3.2. Memory Effect on Seed Morphology in Offspring

**Figure 6:**
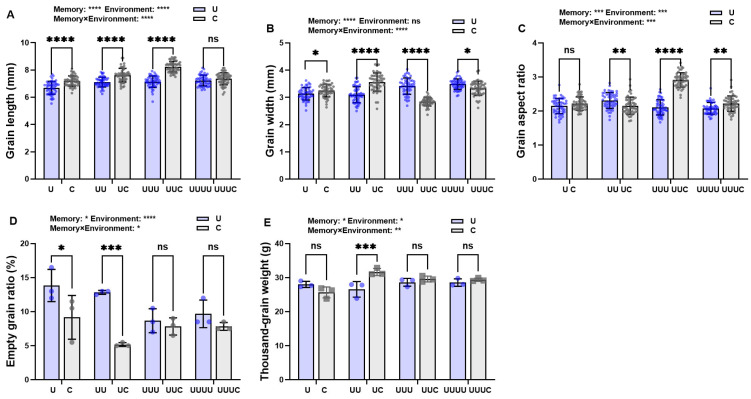
The effects of transgenerational plasticity on seed morphology in different generations of rice. (**A**–**E**) represent grain length, grain width, grain aspect ratio, empty grain ratio, thousand-grain weight under the two treatments in different generations Purple bars represent that the current radiation environment is UV-B and gray bars represent that the current radiation environment is natural light. The horizontal coordinates indicate the different generations (G1, G2, G3, and G4). C and U correspond to generation G1 (where C: natural light treatment, i.e., control; U: one-year UV-B radiation treatment), UC and UU correspond to G2 (where UC: one-year UV-B radiation treatment followed by one-year natural light treatment; UU: two-year continuous UV-B radiation treatment), UUC and UUU correspond to G3 (where UUC: two-year UV-B radiation treatment followed by one-year natural light treatment; UUU: three-year continuous UV-B radiation treatment), and UUUC and UUUU correspond to G4 (where UUUC: three-year UV-B radiation treatment followed by one-year natural light treatment; UUUU: four-year continuous UV-B radiation treatment). * indicates a significant effect (*p* < 0.05), ** indicates a highly significant effect (*p* < 0.01), *** indicates highly significant difference (*p*<0.001), **** indicates very highly significant difference (*p*<0.0001), and ns indicates no significant effects (according to two-way ANOVA).

Within the same generation, when memory and environment were mismatched, grain length under natural light was significantly higher in both G1 and G3 compared with UV-B radiation (Figure 6A). Grain width was significantly higher in the first two generations but decreased in the last two (Figure 6B). The grain aspect ratio, though not significantly different in G1, was significantly lower in G2 and significantly higher in G3 and G4 (Figure 6C). The empty grain ratio was significantly lower in G1 and G2 but had no significant effect in the two radiation environments in the subsequent generations (Figure 6D). The thousand-grain weight was significantly higher in G2 and remained essentially the same in subsequent generations exposed to radiation (Figure 6E).

Radiation memory and environment displayed significant interactions that affected grain length, grain width, grain-length ratio, empty grain ratio, and thousand-grain weight.

#### 2.3.3. Memory Effect on Offspring MDA and Proanthocyanidin Content

**Figure 7:**
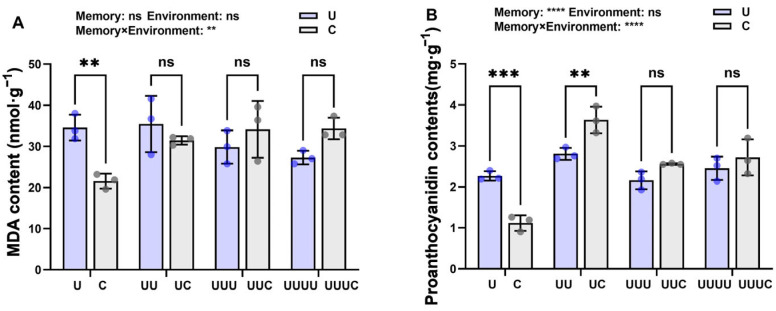
The effects of transgenerational plasticity on MDA and proanthocyanidin content in different generations of rice. (**A**,**B**) represent MDA and proanthocyanidin content under the two treatments in different generations. Purple bars represent that the current radiation environment is UV-B and gray bars represent that the current radiation environment is natural light. The horizontal coordinates indicate the different generations (G1, G2, G3, and G4). C and U correspond to generation G1 (where C: natural light treatment, i.e., control; U: one-year UV-B radiation treatment), UC and UU correspond to G2 (where UC: one-year UV-B radiation treatment followed by one-year natural light treatment; UU: two-year continuous UV-B radiation treatment), UUC and UUU correspond to G3 (where UUC: two-year UV-B radiation treatment followed by one-year natural light treatment; UUU: three-year continuous UV-B radiation treatment), and UUUC and UUUU correspond to G4 (where UUUC: three-year UV-B radiation treatment followed by one-year natural light treatment; UUUU: four-year continuous UV-B radiation treatment). ** indicates a highly significant effect (*p* < 0.01), *** indicates highly significant difference (*p*<0.001), **** indicates very highly significant difference (*p*<0.0001), and ns indicates no significant effects (according to two-way ANOVA).

Over the generations, MDA content decreased when the primary radiation source was UV-B radiation but improved when the radiation environment was natural light. Between different generations, when the radiation environment did not match the radiation memory, the changes in MDA content were not significant (Figure 7A). Proanthocyanidin content showed a tendency to increase and then decrease over the generations. UV-B radiation significantly increased proanthocyanidin content in G1 compared with rice without stress memory. In G2, this parameter was significantly elevated in plants with radiation memory exposed to natural light. In G3 and G4, proanthocyanidin content increased but not significantly (Figure 7B).

Radiation memory had a significant effect on proanthocyanidin content, while the radiation environment had no significant effects on proanthocyanidin content or MDA. The significant interaction between the two affected rice seed growth.

#### 2.3.4. Memory Effect on Enzyme Activity Regulation of Proanthocyanidin Content

**Figure 8:**
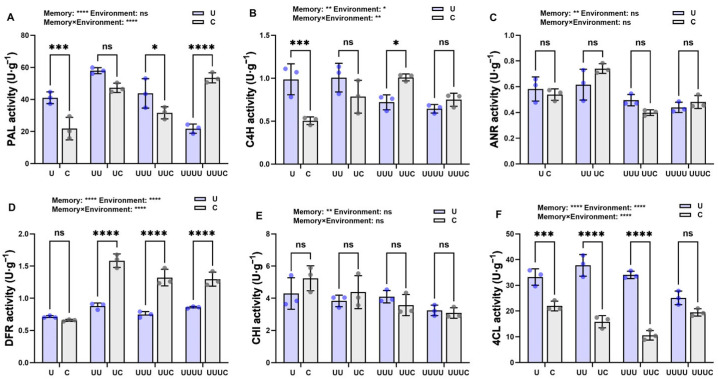
The effects of transgenerational plasticity on enzyme activity in different generations of rice. (**A**–**F**) represent PAL, C4H, ANR, DFR, CHI, and 4CL activity under the two treatments in different generations. Purple bars represent that the current radiation environment is UV-B and gray bars represent that the current radiation environment is natural light. The horizontal coordinates indicate the different generations (G1, G2, G3, and G4). C and U correspond to generation G1 (where C: natural light treatment, i.e., control; U: one-year UV-B radiation treatment), UC and UU correspond to G2 (where UC: one-year UV-B radiation treatment followed by one-year natural light treatment; UU: two-year continuous UV-B radiation treatment), UUC and UUU correspond to G3 (where UUC: two-year UV-B radiation treatment followed by one-year natural light treatment; UUU: three-year continuous UV-B radiation treatment), and UUUC and UUUU correspond to G4 (where UUUC: three-year UV-B radiation treatment followed by one-year natural light treatment; UUUU: four-year continuous UV-B radiation treatment). * indicates a significant effect (*p* < 0.05), ** indicates a highly significant effect (*p* < 0.01), *** indicates highly significant difference (*p*<0.001), **** indicates very highly significant difference (*p*<0.0001), and ns indicates no significant effects (according to two-way ANOVA).

Within the same generation, when the radiation memory and environment were mismatched, PAL activity was significantly decreased under natural light compared with UV-B radiation. In particular, in G2, there was a decrease but no significant difference. In contrast, in G4, the mismatch between environment and memory significantly increased PAL activity (Figure 8A). Although C4H activity was significantly increased by UV-B radiation in generation G1, changes in C4H, ANR, and CHI activity in subsequent generations were not significantly different between the two environmental conditions (Figure 8B,C,E). In the case of memory–environment mismatch, DFR activity was significantly elevated in G2, G3, and G4 (Figure 8D), and 4CL activity significantly decreased (Figure 8F). The two-way ANOVA indicated that radiation memory had a greater effect on the activity of these proanthocyanidin synthases than the environment.

### 2.4. Transcriptome Analysis

#### 2.4.1. Quality Assessment of Sequencing Data

In order to further analyze the differences between rice generations G2 and G3, which were continuously treated with radiation (UU and UUU plants, respectively), the sequencing of six cDNA libraries was performed, and the raw data were filtered to obtain the statistics of clean reads for each sample (see Table A1). The Q30 ratios were all above 95.33%. Then, the high-quality clean reads of the samples were compared with the reference genome (Table A2), and the ratio was above 93.94%, 6.06% of which represented the unmatched ones. The number and proportion of matched rRNAs met the requirements for the next step of the analysis.

#### 2.4.2. Number of Differentially Expressed Genes

**Figure 9:**
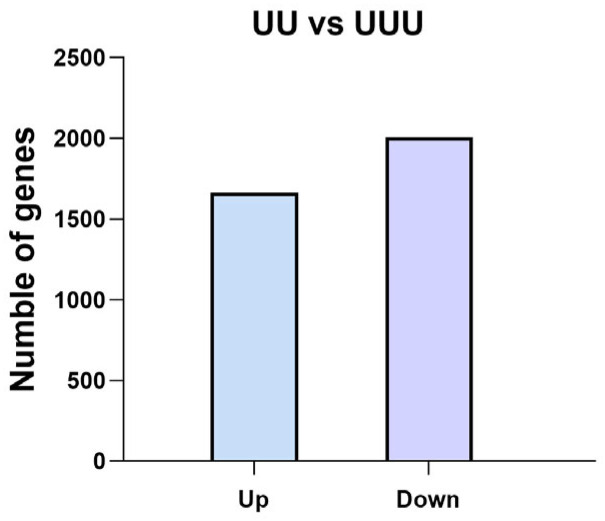
Rice differentially expressed genes in two generations. In the figure, UU and UUU are rice generations treated with UV-B radiation for 2 and 3 consecutive years, respectively.

A total of 3670 differentially expressed genes were identified in rice seeds of generations UU and UUU (Figure 9). Of these, 2007 were down-regulated genes, and 1663 were up-regulated genes. In G3, compared with G2, more genes were down-regulated. These genes may play an important role in how plants of both generations respond to UV-B radiation.

#### 2.4.3. GO Enrichment Analysis

**Figure 10:**
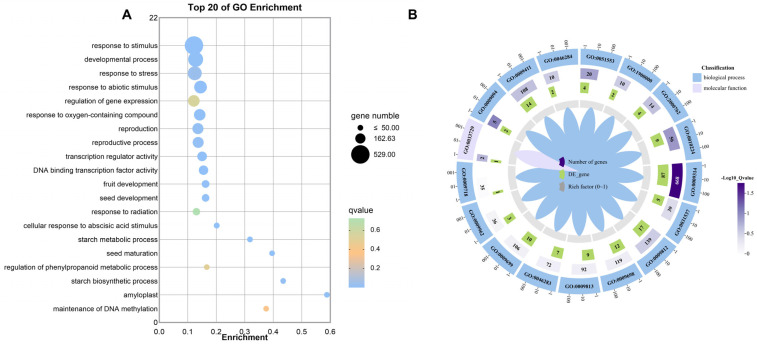
Differentially expressed gene GO enrichment analysis results. (**A**) shows the GO pathway enrichment bubble charts. The horizontal axis represents fold enrichment; the vertical axis represents significant pathways; the size of the solid circles represents the gene number in each pathway; and the color represents the enriched q-value. (**B**) shows the GO enrichment circle diagram of differential genes. The outside lap represents the target GO terms; the middle lap represents the numbers of all genes in GO terms and q-values for gene enrichment for the specified GO term; and the inner lap represents the numbers of differential genes. The ladder column in the center represents the Rich factor of differential genes for each GO term.

According to the top 20 GO enrichment categories for up- and down-regulated genes (Figure 10A), the top three pathways were response to stimuli, developmental process, and response to stress. Regulation of the phenylpropanoid metabolic process, response to radiation, fruit development, and other pathways were also enriched. In particular, maintenance of DNA methylation, regulation of gene expression, and transcription regulator activity were enriched.

The enriched GO terms for the target genes were the pathways of UV stress response, phenylpropane metabolism in the proanthocyanidin synthesis pathway, and the flavonoid synthesis pathway (Figure 10B). By comparing the two different groups of UU and UUU genes, it can be seen that certain differentially expressed genes were enriched in all of these signaling pathways; in particular, the majority were enriched in the pathways of response to radiation and flavonoid metabolic process (Table 2). Moreover, of those genes with significant differences, the majority were down-regulated.

#### 2.4.4. KEGG Enrichment Analysis

**Figure 11:**
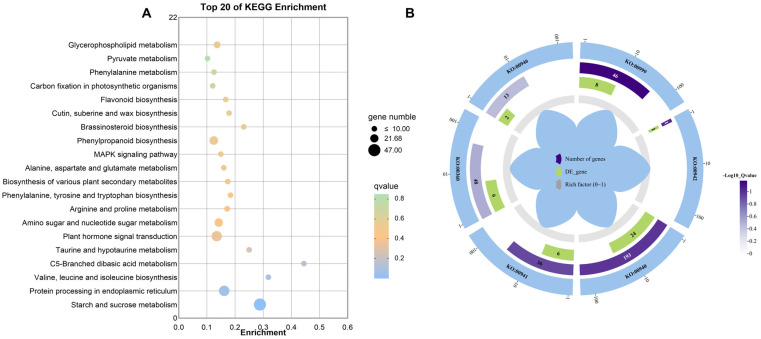
Differentially expressed gene KEGG enrichment analysis. (**A**) shows the KEGG pathway enrichment bubble charts. The horizontal axis represents fold enrichment; the vertical axis represents significant pathways; the size of the solid circles represents the gene number in each pathway; and the color represents the enriched q-value. (**B**) shows the KEGG enrichment circle diagram of differential genes. The outside lap represents the target KEGG terms; the middle lap represents the numbers of all genes in KEGG terms and q-values for gene enrichment for the specified KEGG term; and the inner lap represents the numbers of differential genes. The ladder column in the center represents the Rich factor of differential genes for each KEGG term.

In order to investigate the metabolic pathways involving the differentially expressed genes, they were analyzed for pathway enrichment by using the KEGG database, and the classification statistics relative to two generations, UU and UUU, are shown below. A total of 272 enriched metabolic pathways were analyzed, and the top 20 were selected for counting (Figure 11A). Then, based on the screening of pathways related to proanthocyanidin synthesis, the pathways related to anthocyanin biosynthesis, phenylpropanoid biosynthesis, flavonoid biosynthesis, and phenylalanine metabolism were found. Degradation of flavonoids was also enriched (Figure 11B). Specific information is provided in Table 3. It can be seen that the changes in proanthocyanidin content in both generations under UV-B radiation are related to these pathways.

#### 2.4.5. Screening of Target Genes

After the enrichment analysis of the differentially expressed genes and related functions among the above subgroups, the metabolic functions of these differentially expressed genes were further investigated. Because of their large number, ten genes related to rice proanthocyanidin synthesis and metabolism were screened from the genes differentially expressed between UU and UUU (Table 4).

## 3. Discussion

### 3.1. Effects of Different Intensities of UV-B Radiation on Rice

As an abiotic stressor, UV-B radiation can adversely affect crops such as rice. In this study, UV-B radiation exposure caused decreases in spike weight, grain length, grain width, and thousand-grain weight to different degrees in generation G1. UV-B radiation inhibited grain length more than grain width, and grain morphology changed from long to short. The increase in the rate of empty grains also implied a decrease in the rate of fruiting under UV-B radiation. Spike length directly determines the number and length of spike branches, which in turn affects the number of grains per spike [28]. However, none of the three UV-B radiation intensities had a significant effect on spike length in this experiment. Decreases in these yield parameters might lead to a decrease in rice yield [29]. Rice grain morphology is a complex trait regulated by multiple genes and pathways and, together with thousand-grain weight, is influenced by changes in rice intrinsic physiology and biochemistry in response to UV-B radiation. On one hand, changes in seed phenotype are caused by changes at the cellular level, where UV-B-radiation-induced oxidative stress and DNA damage inhibit cell division, leading to a decrease in cell number [30]. On the other hand, UV-B radiation causes plants to produce more free radicals, which harms the photosynthetic system and reduces dry matter accumulation, thus leading to a decrease in rice thousand-grain weight and an increase in the rate of empty grains [31].

In this experiment, UV-B radiation significantly contributed to elevated proanthocyanidin levels in seeds. Although this parameter decreased under 7.5 kJ·m^−2^ conditions, it was still significantly higher than under natural light. This is because 7.5 kJ m^−2^ exceeds the rice UV-B tolerance threshold, resulting in a decrease in proanthocyanidin content. It can be seen that the response of rice to UV-B is related to radiation intensity. This is consistent with other radiations (e.g., gamma radiation), and the physiological response of plants to radiation and their radiosensitivity should be discussed in relation to the radiation dose [32]. However, procyanidins can still offer protection against UV-B radiation. The phenomenon of smaller rice grains and lower thousand-grain weight but higher proanthocyanidin content under UV-B radiation indicates an alteration in resource allocation, with some energy and nutrients being reallocated for UV-protective substance synthesis. Proanthocyanidins protect the rice seed’s interior from damage [33], further ensuring that seed viability remains constant to maintain reproduction.

### 3.2. Transgenerational Plasticity in Rice in Response to UV-B Radiation

#### 3.2.1. Transgenerational Plasticity Response Characterization

Some colonization-related biological characteristics of plants result in higher survival pressure in the presence of enhanced UV-B radiation [34], and the latter, in the natural environment, is subject to recurrent and long-term changes. Consequently, not only plants’ response to UV-B stress is affected by the current radiation environment; it may also be affected by the mother plant’s radiation environment [35]. In this study, we confirmed that the exposure of successive generations to UV-B radiation altered rice growth. In generations G1 and G2, grain length, grain width, and thousand-grain weight were reduced to different degrees compared with untreated rice, while the empty grain rate and MDA content increased. Clearly, these changes indicate that rice is subjected to oxidative damage from UV-B radiation. However, at the same time, proanthocyanidin content increased significantly and consistently, with high levels being recorded in G1 and G2, a process indicating that the rice plants transmitted stress memory to their progeny. In addition to having effects on rice, prolonged exposure to UV-B radiation was shown to have transgenerational effects on morphology and flavonoid plasticity in *Vicia faba* L. [17]. The behavior whereby the mother plant shares information with the offspring was also documented in *Glechoma longituba* L. subjected to light conditions free of UV-B stress [36], under which plant offspring produced UV defense substances [16]. This is in accord with our study findings.

Rice growth was affected by the exposure of successive generations to UV-B radiation stress, which was mainly noticeable in generation G3, in which compared with G2, the spike weight, grain width, and thousand-grain weight began to increase, while the empty grain ratio, and proanthocyanidin and MDA contents began to decrease. This indicates that the crops began to recover from the growth disruption experienced in the previous period. In G4, plant height, spike weight, grain length, empty grain rate, thousand-grain weight, and MDA content were restored to the control levels, i.e., those in plants exposed to natural light. Further, grain width, tiller number, and biomass also increased, as well as the number of grains per spike, though not significantly. This indicates that transgenerational plasticity improved rice growth in the offspring, and consequently, it enhanced progeny stress tolerance, as also demonstrated in other studies on environmental stress in rice [37]. Based on an overall assessment of rice transgenerational plasticity changes, UV-B radiation exposure altered rice resource distribution across four generations. Given the exposure to UV-B radiation of the previous generation, plants accumulated more UV defenses to mitigate stress and maintain seed-holding activity through the production of high levels of proanthocyanidin content to ensure progeny survival. The increase in progeny total phenolic and antioxidant capacity has also been evidenced in drought-stressed rice [38]. In a later stage, rice growth resumed, and more resources were allocated for growth and reproduction. Thus, rice plants built a tolerance to UV-B radiation. Further study is needed to determine the underlying mechanisms of UV-B resistance.

#### 3.2.2. Mechanism of Transgenerational Changes in Proanthocyanidin Content

In this study, in the four generations, transgenerational plasticity changes in proanthocyanidin levels were significantly correlated with PAL, C4H, 4CL, and DFR activity. The synthesis of proanthocyanidins induced by UV-B radiation is regulated by UVR8 following its interaction with COP1 [39], further leading to the regulation of the expression levels of related genes and the activity of related enzymes [40]. Genes with significant differences in expression levels were identified among genes related to rice proanthocyanidin synthesis and metabolism. C4H enzyme synthesis is regulated by genes such as *OsC4H* and *C4H1*. Most of these genes showed a decrease in expression levels, consistent with changes in physiological proanthocyanidin content in generation G3. These changes were associated with other genes that regulate the flavonoid synthesis pathway (e.g., *OsCCR*), in addition to genes that directly regulate proanthocyanidin synthase expression. Ultimately, this leads to a transgenerational change in proanthocyanidin content. In *Arabidopsis thaliana* L., UV-B radiation exposure led to enhanced expression of genes in the proanthocyanidin pathway, such as *AtCHS* and *AtCHI* [41]. However, in the present study, although the pattern of change in ANR activity across the generations was consistent with that of proanthocyanidin content, the effect on the latter was not significant.

The GO enrichment analysis of the differentially expressed genes revealed that pathways related to seed development, in addition to those related to UV-B radiation response, were enriched. However, it remains to be investigated whether this is related to changes in seed size, empty grain ratio, and thousand-grain weight. Pathways relative to responses to heat, water deficit, temperature, and stimulation were also significantly enriched, suggesting that UV-B radiation increased rice tolerance not only to UV-B radiation but also to stress. Rice seedlings treated with UV-B radiation were previously shown to display enhanced tolerance to NaCl stress [42]. Specifically, the DNA methylation pathway was significantly enriched. In a previous study, reduced levels of DNA methylation in the proximal promoter, intron 1, and intron 2 of maize transcription factor P1 under UV-B stress led to the activation of the R2R3-MYB transcription factor, which is involved in flavonoid synthesis, suggesting a potential role for DNA methylation in the adaptation of local varieties of maize to exposure to high UV-B radiation at high altitudes [43]. However, the changes in rice transgenerational plasticity in proanthocyanidin content across the four generations are related to epigenetic mechanisms and need to be further investigated.

### 3.3. Stress Memory Effect on Transgenerational Plasticity

Stress memory in plants plays a crucial role in their ability to respond to UV-B radiation. The radiation intensity of 7.5 kJ·m^−2^ in this study represents non-lethal UV-B stress that causes damage to a plant but can keep it in a memory-initiated state. Stress memory may persist in rice after the stressor has been removed. In this study, when the offspring with UV-B stress memory were grown under natural light, rice plant height, biomass, tiller number, spike length, and number of grains per spike were significantly lower than when plants were grown under matching memory–environment conditions. The stress memory led to a hysteresis effect after the stressor was removed, and rice growth was significantly inhibited. Despite this, rice still responded to UV-B stress. At this time, the MDA content was elevated, though not significantly, as was proanthocyanidin content. Similarly, it was reported that UV-B-radiation-induced anthocyanin synthesis in *Raphanus sativus* L. maintained an increasing trend for 12 h after stress cessation [44]. Regarding the kernels, the most significant changes were a decrease in empty grain rate and an increase in thousand-grain weight. In a study on the clonal plant *Glechoma longituba* L., it was found that the progeny allocated more resources for defense and increased flavonoid and anthocyanin contents under unpredictable UV-B conditions [45]. Overall, after the stressor was removed, rice experienced negative effects due to stress memory, as the growth strategy was to inhibit growth, allocate more resources to defense and offspring reproduction, increase proanthocyanidin content, and produce fewer but larger seeds.

Some researchers have also suggested that when plants are not subjected to stress for a long period of time, they genetically erase the memory of previous stress conditions to achieve higher environmental adaptability [46]. Plant response to environmental stress follows a hierarchical organization, including physiological, epigenetic, and genetic levels, each of which contributes to stress resistance [47]. Since the intensity of environmental stress determines, to some extent, the degree of plant response, the time of transmission and maintenance of UV-B stress memory may be related to the intensity and duration of UV-B radiation. Furthermore, whether memory persistence after stress elimination is beneficial should also be discussed in this context. By artificially exposing plants to moderate environmental stress, it may be possible to promote stress memory and enhance plant stress tolerance.

In this study, we performed transcriptome analyses for only two generations of rice, and only correlated the changes in transgenerational plasticity from a transcriptional perspective, while the underlying epigenetic mechanisms were not investigated. Future studies may consider the following: (1) Multi-omics could be combined with joint analysis to explore change patterns in plants under UV-B radiation as a whole and then systematically elucidate the interactions and regulatory networks in stress memory. (2) Based on the dual effects of UV-B radiation, how transgenerational plasticity changes under low doses of radiation could be explored. (3) Whether UV-B stress tolerance in seeds and seedlings can be improved without inhibiting growth in rice could be a further research direction.

## 4. Materials and Methods

### 4.1. Overview of Test Site

The experimental site was located at the center of the Yuanyang terraces in Qingkou Village, Xinjie Town, Yuanyang County, Honghe Prefecture, Yunnan Province, China (23°07′ N, 102°44′ E). The altitude of this area is 1600 m, the mean annual temperature is 16.5 °C, and the average annual precipitation is 1410.5 mm. This area is further characterized by the following: background intensity of UV-B radiation, 10 kJ·m^−2^·d^−1^; local soil pH, 5.68; organic matter content, 28.3 g·kg^−1^; total N content, 2.53 g·kg^−1^; total P content, 0.82 g·kg^−1^; total K content, 7.12 g·kg^−1^; alkaline hydrolyzed N, 69.5 mg·kg^−1^; available P, 22.7 mg·kg^−1^; and available K, 168.5 mg·kg^−1^.

### 4.2. UV-B Radiation Treatment

An adjustable lamp holder was placed above each row of rice plants, and a UV-B lamp (40 W and wavelength of 280–320 nm; Shanghai Gucun Instrument Factory, Shanghai, China) was set up to measure the radiation intensity at the top of the plants with a UV-B radiometer (Beijing Normal University Optoelectronic Instrument Factory, Beijing, China); the height of the lamp holder was adjusted according to rice growth. The irradiation time was 7 h per day (10:00–17:00), and no UV-B radiation treatment was performed on cloudy and rainy days to maintain consistency with the treatment group under natural light. The background UV-B intensity at the test site was 10 kJ·m^−2^·d^−1^. Artificially enhanced UV-B radiation treatments at 2.5, 5, and 7.5 kJ·m^−2^ corresponded to 10%, 20%, and 30% of local UV-B radiation, respectively. The rice crops were treated from the booting stage to the end of harvest.

### 4.3. Plant Material and Treatment

The test material was the traditional red rice variety ‘Baijiaolaojing’ from the Yuanyang terraces, which has been cultivated for more than 300 years. The initial seeds (G0), which were not treated with UV-B radiation, were obtained from the Agricultural Science Station in Xinjie Town, China. Field trials were conducted in the Yuanyang terraces. There were 3 plots per treatment; the plot size was 3.9 m × 2.25 m, and the plot interval was 50 cm. A total of 10 rows were planted in each plot, and 10 rice plants were planted per row, where the row spacing was 30 cm and the plant spacing was 15 cm; one seedling was planted per cluster, and 6 and 4 protective rows were planted outside the plots. The average moisture content of the seeds used in the experiment was 11.13%. Seedlings were sown in late February each year. The rice seeds were sown in the leveled field, and when the rice had grown 3 leaves, the better-grown seedlings were selected for transplanting. Seedlings were transplanted manually to a depth of 2 cm into the soil.

G1 plants were obtained by treating G0 seeds with UV-B radiation at different intensities (0 (natural light), 2.5, 5.0, and 7.5 kJ∙m^−2^) in the first year and were harvested in 2020. The radiation intensity that maximized the phenotypic plasticity response index was determined to be 7.5 kJ∙m^−2^. In 2021, G0 and G1 seeds were continuously cultured for 1 year, and G1 and G2 plants were obtained, respectively. G0, G1, and G2 seeds were planted in 2022 to produce G1, G2, and G3 plants and seeds, respectively. Finally, G0, G1, G2, and G3 seeds were sown at the same time on April 23, 2023, and G1, G2, G3, and G4 plants, respectively, were obtained.

**Figure 12:**
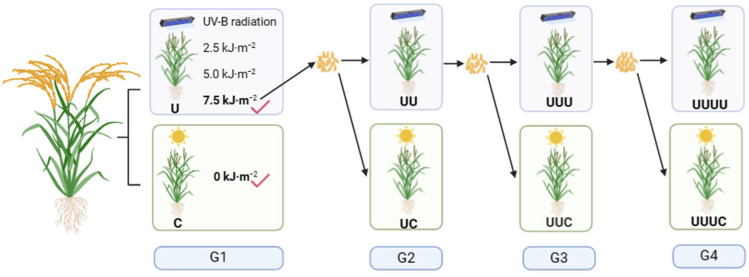
A schematic diagram of the rice treatments. Natural light (0 kJ∙m^−2^) and UV-B radiation treatments (2.5, 5.0, and 7.5 kJ∙m^−2^) were applied in the G1 generation, and the radiation intensity with the largest phenotypic plasticity index (7.5 kJ∙m^−2^) was selected. The original ‘Baijiaolaojing’ seeds were treated with continuous, enhanced UV-B radiation for 1, 2, 3, and 4 years, and natural-light-treated plants were used as controls. G1, G2, G3, and G4 plants were obtained and were named U, UU, UUU, and UUUU or C, UC, UUC, and UUUC, respectively. For example, G1 seeds treated with UV-B radiation were continuously grown for 1 year under 7.5 kJ·m^−2^ UV-B radiation intensity and named UU, while those treated with natural light were named UC.

Natural light and 7.5 UV-B radiation treatments were applied to all generations except G1, which was subjected to four radiation treatments. The treatments receiving UV-B radiation were named U, and those receiving natural light were named C. G0 seeds were grown under 7.5 kJ∙m^−2^ UV-B radiation intensity for four consecutive years to obtain U, UU, UUU, and UUUU plants. UV-B-treated G0, G1, G2, and G3 seeds were also grown under natural light to obtain C, UC, UUC, and UUUC plants. Except for the initial determination of the plasticity response index, the subsequent four generations of plants were planted at the same time. Figure 12 shows a schematic diagram and the nomenclature of the rice treatments.

### 4.4. Indicator Measurements

#### 4.4.1. Growth Traits

Three rice plants were randomly collected from each plot in the maturity stage. The tillers were counted, and plant height was determined by using a scale to determine the height of the rice plant above the root. After harvesting the rice plants, they were placed in an oven, killed at 105 °C for half an hour, and dried at 75 °C to a constant weight; then, the biomass was recorded (the main purpose of killing the plants at 105 °C is to allow the plants to die in the shortest possible time and to minimize the impact on the results of the experiments).

#### 4.4.2. Panicle and Grain Morphological Traits

Forty rice spikes were randomly collected from each experimental plot, with spike length being the distance from the growth point to the tip of the spike, and after determining the spike weight, the grains were threshed to determine the number of grains per spike. Fifty normal grains were randomly taken from each treatment group, placed on a scaled black cardboard, and photographed. Grain length and width were measured by using Image J software version win64, recorded, and averaged, and the length-to-width ratio was calculated. Three samples of 1000 seeds were randomly selected from each plot to determine the mass of 1000 grains, and the empty grain rate = number of empty grains/total number of grains × 100%.

#### 4.4.3. Physiological Traits

Proanthocyanidin content in rice seeds in the maturity stage was determined by using the vanillin method. Briefly, 1.0 g of rice seed was weighed, crushed, and extracted with 20 mL of 70% methanol in an oscillator (300 rpm) for 2 h at room temperature, followed by centrifugation at 8000× *g* for 10 min. After that, 2 mL of the extract was mixed with 5 mL of 1% vanillin–methanol solution (*w*/*v*) and 5 mL of concentrated hydrochloric acid–methanol solution. Next, a sample mix control solution containing 100% methanol was prepared. The solutions were incubated at 30 °C for 20 min away from light; then, the absorbance of the sample and control solutions were measured at 500 nm. The proanthocyanidin content was determined by using a standard catechin (99.8%; Shanghai yuanye Bio-Technology Co., Ltd., Shanghai, China) curve (Y = 0.3097X − 0.0091, R^2^ = 0.9998) as catechin equivalent per gram of sample (mg CE/g). Ten fresh rice spikes were collected from each treatment group in the tasseling stage; the branching peduncle was removed so that only the seeds remained, which were quickly stored in liquid nitrogen for further assays. Malondialdehyde (MDA), phenylalanine ammonialyase (PAL), cinnamate-4-hydroxylase (C4H), anthocyanidin reductase (ANR), dihydroflavonol 4-reductase (DFR), and 4-coumarate:CoA ligase (4CL) content were all assayed by using appropriate kits (Grace Biotechnology, Suzhou, China).

### 4.5. Transcriptome Sequencing

Based on the measurements of traits under different UV-B radiation intensities, we found that these traits were the most plastic under UV-B radiation at 7.5 kJ∙m^−2^. Rice plants of generations G2 and G3 grown under this radiation intensity were selected for RNA sequencing. The tissue samples used in this experiment were taken from rice seeds in the tasseling stage. For each treatment, three independent biological replicates were randomly selected. To extract RNA from tissue samples, the latter were ground into powder in liquid nitrogen.

Total RNA was extracted by using a Trizol kit (Invitrogen, Carlsbad, CA, USA). RNA quality was assessed using an Agilent 2100 Bioanalyzer (Agilent Technologies, Santa Clara, CA, USA) and evaluated with RNA-free agarose gel electrophoresis.

Standard library construction procedures were followed, and mRNA purified from total RNA was used. These procedures included mRNA purification and fragmentation, double-stranded cDNA synthesis, double-stranded cDNA purification and repair, junction ligation, ligation product purification and fragment size sorting, PCR, and quality assessment. A total of 6 libraries (UU treatment of G2 × 3 biological replicates) + (UUU treatment of G3 × 3 biological replicates) were sequenced on the Illumina Novaseq6000.

The quality assessment of the raw data by using pairs of the sequenced raw data was performed with FastQC, and quality clipping was performed with Trimmomatic version 0.36 to obtain relatively accurate valid data. The latter were compared to the reference genome by using HISAT2 to statistically map information. Gene expression was assessed by using StringTie version 1.3.3b and known gene models, and the relative expression of genes was measured by using TPM (Transcripts Per Million) to measure the proportion of a particular transcript in the RNA pool. This was followed by analysis with DESeq2. Genes were screened for significant differences with q-value ≤ 0.05 and multiplicity of differences |FoldChange| ≥ 2, as well as between groups by comparing the expression values that were ≥5 in at least one sample or group. GO and KEGG enrichment results were analyzed by using clusterProfiler version 3.0.5.

### 4.6. Plasticity Response Index

According to the calculation method by Shuo Wang [48], the plasticity response index is determined as follows: *PPI* = (*P*_U_ − *P*_C_)/*P*_C_, where *P*_U_ and *P*_C_ denote the average value of each index under UV-B radiation and natural light, respectively. The value of *PPI* is taken as an absolute value, and its magnitude is positively correlated with the magnitude of phenotypic plasticity. The response index of morphological plasticity related to UV-B radiation in nth-generation rice is as follows: *PPI*_Gn_ = *PPI*_grain length_ + *PPI*_grain width_ + *PPI*_thousand grain weight_ + …… + *PPI*_proanthocyanidin content._

### 4.7. Data Analysis

SPSS 23 statistical software was used to test the significance of the differences between treatments. One-way ANOVA was performed with the Duncan test at *p* < 0.05, and two-way ANOVA was performed by using Bonferroni correction for post hoc multiple comparisons at *p* < 0.05. Graphs were prepared using GraphPad version 9.4.1.

## 5. Conclusions

In the face of recurrent UV-B radiation stress, rice achieves adaptive transgenerational plasticity mainly by adjusting resource allocation for defense and developmental reproduction purposes. In this study, on the one hand, higher levels of proanthocyanidin content were maintained in generations G1 and G2, while on the other hand, reproductive allocation was increased in generations G3 and G4.

## Figures and Tables

**Table 1:** Effect of UV-B radiation on seeds and proanthocyanidins of generation G1.

Treatment	Spike Length/cm	Spike Weight/g	Grain Length/mm	Grain Width/mm	Thousand-Grain Weight/g	Empty Grain Ratio/%	Proanthocyanidin Content /mg·g^−1^	*PPI*_G1_
0 kJ·m^−2^	26.09 ± 0.32 a	5.59 ± 0.26 a	7.50 ± 0.48 a	3.21 ± 0.23 b	28.75 ± 0.13 a	8.38 ± 0.66 c	1.12 ± 0.19 c	
2.5 kJ·m^−2^	25.87 ± 0.39 a	4.70 ± 0.23 b	7.07 ± 0.35 b	3.44 ± 0.25 a	26.33 ± 0.24 b	10.13 ± 0.24 b	1.84 ± 0.26 b	1.59
5 kJ·m^−2^	25.91 ± 0.30 a	5.06 ± 0.25 ab	6.96 ± 0.42 b	3.12 ± 0.25 c	26.03 ± 0.26 b	11.50 ± 0.58 b	2.63 ± 0.38 a	1.09
7.5 kJ·m^−2^	26.44 ± 0.37 a	5.00 ± 0.19 ab	6.39 ± 0.39 c	2.95 ± 0.23 d	24.68 ± 015 c	17.13 ± 0.47 a	2.27 ± 0.41 ab	2.06

Note: The values 0, 2.5, 5, and 7.5 indicate different UV-B radiation intensities. The data in the graphs represent means ± standard deviations, and different letters in the same column indicate significant differences between treatments (according to one-way ANOVA with Duncan’s test; *p* < 0.05).

**Table 2:** Enrichment of target GO terms.

GO Term	Description of GO Term	Genes	Up	Down
GO:0051553	flavone biosynthetic process	4	2	2
GO:1900000	regulation of anthocyanin catabolic process	2	0	2
GO:2000762	regulation of phenylpropanoid metabolic process	14	4	10
GO:0010224	response to UV-B	9	4	5
GO:0009314	response to radiation	87	42	45
GO:0031537	regulation of anthocyanin metabolic process	5	1	4
GO:0009812	flavonoid metabolic process	17	6	11
GO:0009698	phenylpropanoid metabolic process	12	1	11
GO:0009813	flavonoid biosynthetic process	9	4	5
GO:0046283	anthocyanin-containing compound metabolic process	7	3	4
GO:0009699	phenylpropanoid biosynthetic process	10	2	8
GO:0009962	regulation of flavonoid biosynthetic process	3	1	2
GO:0009718	anthocyanin-containing compound biosynthetic process	1	1	0
GO:0033729	anthocyanidin reductase activity	1	0	1
GO:0009094	L-phenylalanine biosynthetic process	2	1	1
GO:0009411	response to UV	14	7	7
GO:0046284	anthocyanin-containing compound catabolic process	2	0	2

**Table 3:** Enrichment of target pathways.

KO Term	Description of GO Term	Differential Genes	Up	Down
KO:00999	Biosynthesis of various plant secondary metabolites	8	4	4
KO:00942	Anthocyanin biosynthesis	1	1	0
KO:00940	Phenylpropanoid biosynthesis	24	7	17
KO:00941	Flavonoid biosynthesis	6	2	4
KO:00360	Phenylalanine metabolism	6	1	5
KO:00946	Degradation of flavonoids	2	1	1

**Table 4:** UV-B-radiation-responsive genes related to proanthocyanidin synthesis and metabolism.

Gene ID	Gene Name	Changes	Description
Os02g0467000	*OsC4H*	down	putative cinnamate 4-hydroxylase
Os10g0578950	*OsACS2*	down	4-coumarate-CoA ligase-like 2
Os08g0448000	*Os4CL5*	down	probable 4-coumarate-CoA ligase 5
Os08g0441500	*OsCCR*	down	cinnamoyl-CoA reductase 1
Os06g0623200	*OsCCR28*	down	cinnamoyl-CoA reductase 2
Os03g0112900	*OsF5HL2*	down	cytochrome P450 84A1
Os02g0467600	*C4H1*	down	cytochrome P450 CYP73A100
Os02g0767300	*FLS*	up	flavonol synthase/flavanone 3-hydroxylase
Os05g0578500	*OsCCR23*	down	cinnamoyl-CoA reductase-like SNL6

## Data Availability

The original contributions presented in the study are included in the article. Further inquiries can be directed to the corresponding authors.

## Appendix A

**Table A1:** Statistical table after data filtering.

Sample	Clean Data (bp)	AF_Q20 (%)	AF_Q30 (%)	AF_N (%)	AF_GC (%)
UU1	7,215,007,346	98.85%	95.48%	0.00%	51.76%
UU2	7,386,387,904	98.82%	95.33%	0.00%	50.99%
UU3	6,857,180,340	99.00%	95.94%	0.00%	51.16%
UUU1	7,621,922,903	98.90%	95.72%	0.00%	53.06%
UUU2	7,813,042,637	98.94%	95.86%	0.00%	51.42%
UUU3	7,536,985,572	99.02%	96.16%	0.00%	51.40%

Note: AF_Q20 and AF_Q30 represent the ratio of bases with quality not less than 20 and 30 in the filtered clean reads; AF_N represents the ratio of indeterminate bases in the filtered clean reads; and AF_GC represents the ratio of the number of bases G and C in the total number of bases in the filtered clean reads. UU1, UU2, and UU3 represent three biological replicates of G2 generation rice grains under continuous UV-B radiation treatment; UUU1, UUU2, and UUU3 represent three biological replicates of G3 generation rice grains under continuous UV-B radiation treatment.

**Table A2:** Statistical results of a sample-reference genome comparison.

Sample	Total	Unmapped	Unique Mapped	Multiple Mapped	Total Mapped
UU1	48,933,326	2,874,464 (5.87%)	44,715,560 (91.38%)	1,343,302 (2.75%)	46,058,862 (94.13%)
UU2	47,575,544	1,840,318 (3.87%)	41,360,594 (86.94%)	4,374,632 (9.20%)	45,735,226 (96.13%)
UU3	50,172,210	2,042,037 (4.07%)	42,408,808 (84.53%)	5,721,365 (11.40%)	48,130,173 (95.93%)
UUU1	48,233,244	2,366,783 (4.91%)	43,710,529 (90.62%)	2,155,932 (4.47%)	45,866,461 (95.09%)
UUU2	51,403,966	2,769,362 (5.39%)	47,184,683 (91.79%)	1,449,921 (2.82%)	48,634,604 (94.61%)
UUU3	52,697,298	3,191,709 (6.06%)	47,900,776 (90.90%)	1,604,813 (3.05%)	49,505,589 (93.94%)

Note: Sample: sample name, Total: the total number of reads after filtering, Unmapped: the number and proportion of rRNA reads not compared with the total number, Unique_Mapped: the number and proportion of single rRNA reads compared with the total number, Multiple_Mapped: the number and proportion of multiple rRNA reads corresponding to the total number, Total_Mapped: the number and proportion of rRNA reads compared with the total number. UU1, UU2, and UU3 represent three biological replicates of G2 generation rice grains under continuous UV-B radiation treatment; UUU1, UUU2, and UUU3 represent three biological replicates of G3 generation rice grains under continuous UV-B radiation treatment.
